# Refractory Chronic Kidney Disease–Associated Pruritus: Old Remedies and Novel Agents

**DOI:** 10.1016/j.xkme.2026.101315

**Published:** 2026-03-11

**Authors:** Kelly Chenlei Li, Brendan Smyth, Mark Brown, Mehwish Asif, Frank Brennan

**Affiliations:** 1 Faculty of Medicine and Health, UNSW, Sydney, Australia; 2 Department of Renal Medicine, St George Hospital, Kogarah, NSW, Australia; 3 NHMRC Clinical Trials Centre, University of Sydney, Camperdown, NSW, Australia

**Keywords:** CKDaP, difelikefalin, lignocaine, quality of life, uremic pruritus

## Abstract

Refractory chronic kidney disease associated pruritus (CKDaP) has limited therapeutic options, leading to significant distress, profound sleep disturbance, and reduced quality of life. We describe 2 cases of severe CKDaP in which conventional treatments were unsuccessful and novel therapeutic strategies were employed. The first case involves a patient receiving hemodialysis who achieved significant symptom relief with a subcutaneous lignocaine infusion, delivered using a syringe driver over 2 weeks followed by oral mexiletine. The second case concerns a kidney failure patient managed on the conservative kidney management pathway, who experienced marked improvement following weekly intravenous difelikefalin despite not receiving chronic hemodialysis. These cases underscore the challenges faced by patients and clinicians in managing severe CKDaP and its profound impact on quality of life and emotional distress. Furthermore, these cases highlight the variability in individual treatment responses and the therapeutic potential of repurposed medicines and emphasize the need for further research in this area.

Chronic kidney disease associated pruritus (CKDaP) is a frequent and debilitating symptom in patients with kidney failure. Severe itch affects around 25% of patients receiving dialysis and patients with nondialysis CKD, and is associated with adverse clinical outcomes including impaired sleep, anxiety and depression, poorer quality of life, and increased hospitalization.^1^, ^2^, ^3^, ^4^, ^5^

Numerous treatments for CKDaP have been reported, the majority with lower levels of evidence.^6^^,^^7^ A Cochrane review found that γ-aminobutyric acid (GABA) analogs, including gabapentin and pregabalin, were the most studied and had the greatest reduction in itch scores.^8^ Subsequent randomized control trials have further demonstrated good efficacy and tolerability of difelikefalin, a κ-opioid agonist.^9^ Beyond these treatments, however, therapeutic options for CKDaP remain limited, especially in severe and refractory cases.

We describe 2 cases of CKDaP in which patients suffered overwhelming itch despite multiple lines of therapies and experienced significant distress from their symptoms. Improvement in symptoms was achieved with off-label use of lignocaine and difelikefalin, respectively. These cases highlight the need for new therapies, which may originate from renewed interest in, or novel use of, existing drugs.

## Case 1

A 65-year-old woman with kidney failure secondary to diabetic kidney disease was receiving hemodialysis (HD) for 4 years. Severe itch, reported using iPOS-renal, started 6 months before dialysis initiation at an estimated glomerular filtration rate (eGFR) of 14 mL/min/1.73 m^2^ and persisted the initiation of HD. Integrated Palliative Care Outcome Scale (IPOS)-renal is a patient-reported outcome measure using a short questionnaire of 15 symptoms, each rated as none, mild, moderate, severe, or overwhelming, validated in advanced kidney disease populations.^10^

In addition to skin emollients, treatments sequentially included pregabalin 75 mg daily, evening primrose oil oral capsules 2 daily, initiation of extended hours nocturnal dialysis using high-flux membrane and hemodiafiltration to optimize clearance (6 hours, 3 times weekly, Kt/V > 2), oral cetirizine 10 mg daily, and topical compound ointment (menthol 5%, lignocaine 7%, and capsaicin 0.025%). These treatments achieved only partial responses; itch remained moderate/severe (iPOS). Difelikefalin at 0.5 μg/kg 3 times weekly, when it became available in our institution, reduced itch to mild for 18 months. It was subsequently uptitrated to 1 μg/kg, with a further 5 months of good symptom control. Biochemistry on HD, apart from a short period of acute illness, was within optimal dialysis targets, including a Ca level of 8.0-10.4 mg/dL, PO_4_ level of 3.72-6.19 mg/dL, and parathyroid hormone (PTH) level of <280 pg/mL.

**Figure 1:**
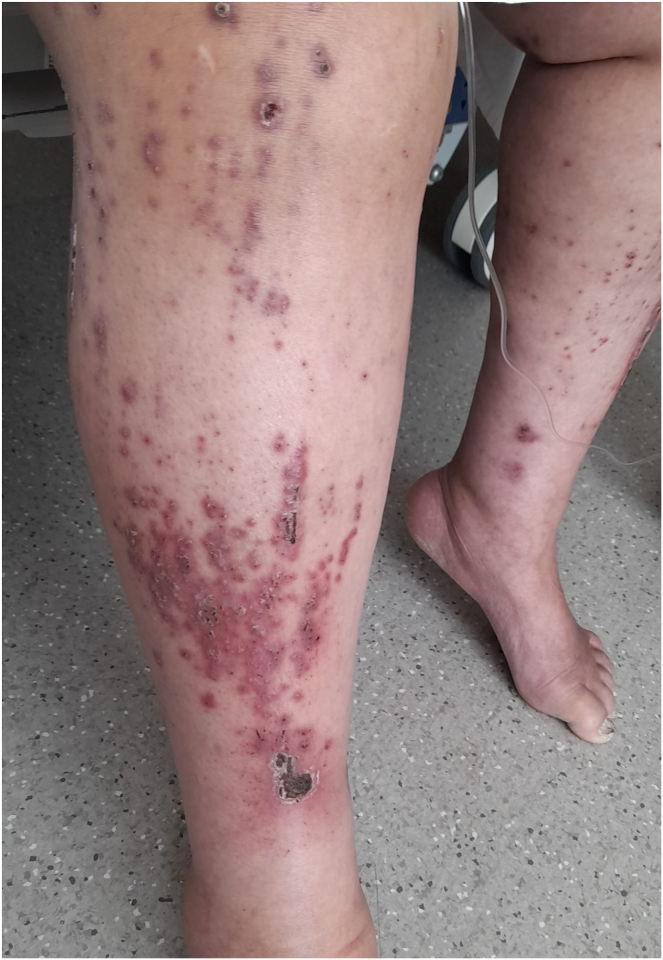
Skin changes of CKDaP because of ferocious scratching. Note: Skin trauma as a result of excessive scratching.

Thereafter, the patient developed an acute exacerbation of itch, reported as overwhelming (iPOS), affecting her entire body, associated with severe sleep disturbance, ferocious scratching with Koebner’s lines (Figure 1), and emotional distress. At this time, predialysis Ca and PO_4_ levels were 9.14 mg/dL and 4.46 mg/dL, respectively. She remained on skin emollients, pregabalin 75 mg taken at night, and difelikefalin 1 μg/kg 3 times weekly and received an empirical course of ivermectin (withdrawn when skin scraping for scabies was negative). Skin biopsy showed spongiotic dermatitis with surface impetiginization and negative immunofluorescence, and dermatology assessment confirmed the diagnosis of CKDaP.

The patient was administered a continuous subcutaneous infusion (CSCI) of lignocaine at 200 mg/24 hours, with uptitration to 600 mg/24 hours within 48 hours. Lignocaine (500 mg/5 mL) was delivered via a syringe driver, a small, battery-operated device delivering medications continuously over 24 hours through a subcutaneously needle, with dosing adjusted and loaded daily. The patient was monitored clinically for side effects, in accordance with local protocol (Supplementary Table S1). Itch had almost completely resolved by day 4, but withdrawal of lignocaine on day 5 led to an acute surge in itch on day 9, and lignocaine was readministered, with a good result (iPOS mild) 48 hours later. On day 14, lignocaine was weaned, and the patient was transitioned successfully to oral mexiletine 150 mg twice daily. Four months later, itch score remained mild (iPOS).

## Case 2

An 86-year-old woman with kidney failure secondary to diabetic kidney disease, eGFR 9 mL/min/1.73 m^2^ managed with conservative kidney management (CKM) without dialysis, presented with severe, prolonged, treatment-resistant itch because of CKDaP for over 6 months. Prior therapies, including skin emollients, topical corticosteroid, topical compound ointment (lignocaine 10%, menthol 10%, capsaicin 0.05%), pregabalin 25 mg daily, mirtazapine 30 mg daily, oral turmeric, ultraviolet B phototherapy (18 sessions), and lignocaine up to 1200 mg/day via CSCI, provided no relief. She reported itch as 10 out of 10 on the Worst Itch Numerical Rating Scale (WI-NRS) and 25 out of 25 on the 5D Pruritus Scale and rated both itch and sleep disturbance as overwhelming on iPOS-Renal.^10^, ^11^, ^12^ Biochemistry results included a Ca level of 11.4 mg/dL, PO_4_ level of 5.02 mg/dL, and PTH level of 305 pg/mL.

**Figure 2:**
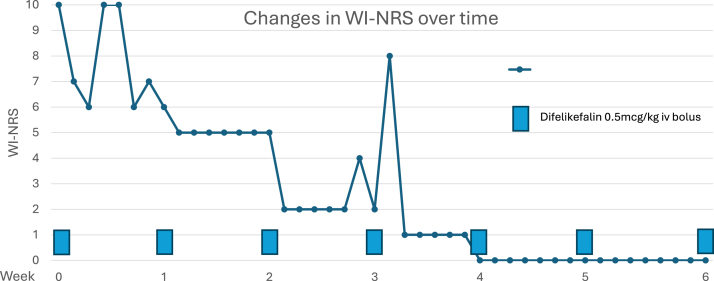
Changes in itch severity after commencing intravenous difelikefalin. Abbreviations: WI-NRS, Worse Itch Numerical Rating Scale.

Permission was obtained from the local institutional drug committee for intravenous difelikefalin, indicated only for use in HD patients with CKDaP. The patient was administered intravenous difelikefalin 0.5 μg/kg bolus at weekly intervals. By week 3, WI-NRS improved from 10 to 2 out of 10, with complete symptom resolution (WI-NRS 0/10) by week 4. In addition, 5D scores similarly improved from 25 to 5 out of 25 as had sleep, all sustained through to week 10. Daily WI-NRS was used in the initial 6 weeks to assess for required dosing frequency, and no reduction in drug efficacy was observed in the days before scheduled dosing (Figure 2). No adverse effects were reported.

Thereafter, the patient was hospitalized because of an unrelated illness and developed worsening itch, likely a result of antimicrobials, opioid analgesia, and other concomitant medications introduced at the time. Itch severity returned to baseline WI-NRS 0-2 after discharge 3 weeks later. At 6 months, the patient remains stable on CKM, and her eGFR is unchanged at 9 mL/min/1.73 m^2^, with a Ca level of 10.3 mg/dL and PO_4_ level of 5.05 mg/dL. She continues weekly intravenous difelikefalin with excellent symptom control: WI-NRS 0-1, none to mild itch on iPOS, and 5D 5-7 out of 25.

## Discussion

CKDaP is a challenging condition to manage, and severe cases can be extremely distressing for patients. A variety of therapies have been used by different clinicians worldwide, many of which have only a low level evidence of efficacy and are not well studied.^7^

The pathophysiology of CKDaP is complex, incompletely understood, and thought to involve nonhistamine dependent pruritic pathways, with multiple other pruritogenic cytokines (eg, IFN-γ, IL-6, TNF-α) implicated.^6^ Proposed mechanisms include altered opioid receptor activity, systemic and microinflammation of the skin, immune dysregulation, and neuronal injury, leading to complex crosstalk among dermal mast cells, keratinocytes, Th-1 lymphocytes, and nerve fibers, some of which become targets for therapy.^6^^,^^13^

Both cases described the use of lignocaine for CKDaP. Lignocaine and mexiletine block sodium channels in neuronal cell membranes and are local anesthetic agents widely and safely administered systemically to relieve neuropathic pain.^14^ In palliative care settings, lignocaine can be administered as a CSCI for cancer pain and to treat severe and intractable itch in cutaneous T cell lymphoma, another condition, like CKDaP, associated with nonhistamine-dependent pruritic pathways.^15^^,^^16^ Parenteral lignocaine on HD has been trialed to treat itch and found to be efficacious as early as 1977, but it has not been routinely used for this indication since.^17^

In case 1, CSCI lignocaine was administered at doses comparable to those used for cancer pain at our institution. It was used successfully and safely in this patient, although early discontinuation led to a relapse of itch. Mexiletine, an oral medication with the same mechanism of action, was substituted. This class of medications may provide a future therapeutic option for intractable itch in CKDaP, although well designed studies are needed. The lack of efficacy in case 2 highlights the importance of interindividual differences in response to therapy in CKDaP, likely representing the operation of multiple pathways transmitting itch that vary in prominence between individuals. Prospective studies comparing different treatment pathways are needed to better subtype CKDaP such that time spent on ineffective individual therapeutic trials is minimized.

Case 2 highlights the use difelikefalin in a non-HD patient. Difelikefalin is a peripherally restricted κ-opioid agonist and its intravenous formulation has good evidence for efficacy and tolerability in HD patients, but has limited data outside of the HD population.^9^ Given the shared pruritus mechanisms in kidney failure, data from HD population were extrapolated for the use of difelikefalin in this CKM patient with an eGFR of 9 mL/min/1.73 m^2^. Difelikefalin is usually dosed at 0.5 μg/kg as a bolus 3 times weekly after HD.^9^ A pharmacokinetic study showed that difelikefalin is predominantly renally excreted (80.5%) in subjects with normal kidney function, but in kidney failure, 58.8% of the dose was recovered in feces, with the remainder removed by HD (19.5%) and residual kidney function (11.2%).^18^ Weekly dosing as an intravenous bolus at 0.5 μg/kg was administered to this patient, taking into account the low eGFR and the absence of drug removal by HD, as well as the practicality of treatment. For this patient, weekly intravenous difelikefalin was found to be efficacious, practical, and safe.

Repurposing established medications to alleviate complex and distressing symptoms, when limited therapies are available, is common practice in palliative care.^19^ Careful considerations of underlying disease pathophysiology, drug formulation, pharmacokinetic properties, and established clinical efficacy and side effects profile are required.

These 2 cases highlight multiple issues in the care of those with CKDaP: (1) the profound impact of severe pruritus in terms of distress and poor quality of life, and (2) the interindividual variability in response to therapy that frequently results in patients cycling through multiple different therapies. They also demonstrate the potential that repurposed pharmaceuticals may offer in severe and refractory CKDaP. Prospective studies are required to explore the possible subphenotypes of CKDaP, provide evidence to guide treatment sequencing, and test whether novel treatment approaches, such as used in these cases, warrant more widespread use.
