# Supplementary material for: Refractory Chronic Kidney Disease–Associated Pruritus: Old Remedies and Novel Agents
Source: Kidney Med. 2026 Mar 11;8(5):101315. doi: 10.1016/j.xkme.2026.101315 (PMC13099442; doi:10.1016/j.xkme.2026.101315)
Supplement: Supplementary File (PDF) — Table S1 [file mmc1.pdf]

Medicine Guideline

Subcutaneous lidocaine (lignocaine) for refractory neuropathic pain in the Palliative Care Setting

Health
South Eastern Sydney
Local Health District

**Areas where Protocol/Guideline applicable**	SESLHD Inpatient settings (including Calvary hospital)
**Authorised Prescribers:**	Specialist Palliative Care Services
**Indication for use**	Must be used under the supervision of a Palliative Care Specialist: -<br>1. Refractory neuropathic pain not responding to standard analgesic drugs, including optimal use of opioids and adjuvant therapies.<br>2. Refractory pruritis when the oral route is no longer available.
**Clinical condition**	All conditions causing refractory neuropathic pain or pruritus as per the indications for use.
**Proposed Place in Therapy**	Lidocaine is a systemic local anaesthetic agent and known membrane stabiliser. It is used in the palliative care setting as a third or fourth line drug in the treatment of complex & refractory neuropathic pain.
**Adjunctive Therapy**	Can be used alone or in conjunction with other neuropathic pain agents depending on the clinical circumstances.
**Contraindications**	• Adams-Stokes syndrome, Wolff-Parkinson-White syndrome<br>• Severe atrioventricular, sino-atrial or intraventricular heart block not managed with a pacemaker<br>• Sensitivity to amide-type local anaesthetics<br>• Patients on flecainide
**Precautions and Relative Contra-indications**	**Cardiac monitoring in the palliative care setting is not indicated due to doses not exceeding the threshold of 2,800mg (2.8g) over 24 hours via CSCI.**<br><br>Use with caution in patients with known cardiac disease, cerebral palsy, and history of arrhythmia. Where possible, aim to correct electrolyte balances prior to commencement.<br><br>Consider dose modification (up to 50%) in renal or hepatic impairment, and in the context of frailty.
**Important Drug**	Avoid in patients taking flecainide

<table>
<tr><td>Interactions</td><td></td></tr>
<tr><td>Dosage</td><td>Lidocaine has a narrow therapeutic index and dose is determined by consultation with Palliative Care Specialist.

Starting dose:
Lidocaine 0.5 mg/kg/hr (i.e. 200 to 800 mg over 24 hours) via CSCI[6]. Based on ideal body weight.

Titration:
Increase by 200 to 800 mg every 24 hours as required; titrate to effect.

Maximum dose 2,800 mg (2.8g) per 24 hours (approximately 120 mg/hr)

Consider dose modification (up to 50%) in renal or hepatic impairment, and in the context of frailty.</td></tr>
<tr><td>Prescribing Instructions</td><td>Lidocaine must be prescribed on the eMM system. In the absence of eMM systems, the appropriate paper medication chart may be used.</td></tr>
<tr><td>Administration Instructions</td><td>
<table>
<tr><td>DOSE of lidocaine</td><td colspan="2">VOLUME & recommended FORMULATION of lidocaine</td><td>Approx. Volume of Water for Injection (WFI) to make total volume</td></tr>
<tr><td></td><td>Lidocaine 2%</td><td>Lidocaine 10%</td><td>WFI</td></tr>
<tr><td>200mg</td><td>10mL</td><td>-</td><td>7 mL</td></tr>
<tr><td>400mg</td><td>-</td><td>4 mL</td><td>13 mL</td></tr>
<tr><td>500mg</td><td>-</td><td>5 mL</td><td>12 mL</td></tr>
<tr><td>600mg</td><td>-</td><td>6 mL</td><td>11 mL</td></tr>
<tr><td>700mg</td><td>-</td><td>7 mL</td><td>10 mL</td></tr>
<tr><td>800mg</td><td>-</td><td>8 mL</td><td>9 mL</td></tr>
<tr><td>900mg</td><td>-</td><td>9 mL</td><td>8 mL</td></tr>
<tr><td>1000mg</td><td>-</td><td>10 mL</td><td>7 mL</td></tr>
<tr><td>1100mg</td><td>-</td><td>11 mL</td><td>6 mL</td></tr>
<tr><td>1200mg</td><td>-</td><td>12 mL</td><td>5 mL</td></tr>
<tr><td>1300mg</td><td>-</td><td>13 mL</td><td>4 mL</td></tr>
<tr><td>1400mg</td><td>-</td><td>14 mL</td><td>3 mL</td></tr>
<tr><td>1500mg</td><td>-</td><td>15 mL</td><td>2 mL</td></tr>
<tr><td>1600mg</td><td>-</td><td>16 mL</td><td>1 mL</td></tr>
</table>

For doses < 1600 mg; use a 20 mL syringe and make the volume up</td></tr>
</table>

	to 17 mL.<br><br>Doses > 1600 mg will require a 30 mL syringe
**Preparations**	Lidocaine (lignocaine) 2% 100 mg/5 mL ampoules<br><br>Lidocaine (lignocaine) 10% 500 mg/5mL ampoules
**Drug Compatibility**	Lidocaine should not be mixed in a syringe with any other medication due to lack of robust compatibility data. Lignocaine may be given in conjunction with ketamine but NOT in same syringe driver.
**Adverse effects**	Monitor closely for the following **initial signs** of systemic toxicity:<br>• Light-headedness, Dizziness<br>• Perioral numbness or tingling (around lips)<br>• Tinnitis<br>• Metallic taste<br>• Drowsiness and dysarthria.<br><br>If any of the above are observed, cease infusion immediately and inform Palliative Care Medical Officer. Lidocaine infusion may be restarted at a lower dose.<br><br>**Worsening toxicity is indicated by the progressive appearance of:**<br>• Visual changes<br>• Muscle spasm<br>• Seizures<br>• Coma<br>• Cardiorespiratory depression and arrest
**Monitoring requirements**<br>Safety<br>Effectiveness (state objective criteria)	Monitor for signs of adverse effects (as above) and if any of the initial signs of toxicity occur cease the infusion and report to the Palliative Care consultant immediately.<br><br>Perform 4-hourly subcutaneous infusion site checks as per Subcutaneous Syringe Driver inpatient management form SES130.021. These paper forms need to be purchased as per local processes.
**Practice Points**	Lidocaine is only given by continuous subcutaneous infusion via syringe driver. It is NOT to be given by intermittent bolus subcutaneous injections.
**Management of Complications**	If patient reports side effects, the infusion should be ceased and management discussed with palliative care team. Where appropriate, consider an ECG after discussion with the team.

**Basis of Protocol/Guideline:**	1. Benowitz NL, Meister W. Clinical pharmacokinetics of lignocaine. Clinical pharmacokinetics. 1978;3(3):177-201<br>2. Pasero CM, M. Pain Assessment and Pharmacologic Management. Missouri: Mosby Elselvier; 2010<br>3. Schwartzman RJ, Patel M, Grothusen JR, Alexander GM. Efficacy of 5-day continuous lidocaine infusion for the treatment of refractory complex regional pain syndrome. Pain medicine (Malden, Mass). 2009;10(2):401-12<br>4. Swenson BR, Gottschalk A, Wells LT, Rowlingson JC, Thompson PW, Barclay M, et al. Intravenous lidocaine is as effective as epidural bupivacaine in reducing ileus duration, hospital stay, and pain after open colon resection: a randomized clinical trial. Regional anesthesia and pain medicine. 2010;35(4):370-6.<br>5. Hsu Y-W, Somma J, Newman M, Mathew JP. Population Pharmacokinetics of Lidocaine Administered During and After Cardiac Surgery. Journal of cardiothoracic and vascular anesthesia. 2011;25(6):931-6.<br>6. Palliative Care Formulary online. In Medicines Complete. Pharmaceutical Press. Available via CIAP accessed April 2025.<br>7. Palliative Care [December 2024]. In Therapeutic Guidelines Ltd. Available via CIAP, accessed April 2025.<br>8. CHCK Policy 'Pain Management (Neuropathic – Lignocaine & Ketamine). September 2018<br>9. Dickman A, Schneider J. 2016. The Syringe Driver: Continuous subcutaneous infusions in palliative care. Oxford University Press; 2016<br>10. Macleod, R Macfarlane, S. 2018 The Palliative Care Handbook. 9th Ed. Hammondcare Media.
**Groups consulted in development of this guideline**	St George Palliative Care Team<br>SESLHD Palliative Care working party<br>Dr Caitlin Sheehan, Staff Specialist St George & Calvary Hospital

**AUTHORISATION**	
Author (Name)	Dr Linda Sheahan
Position	SESLHD Stream Director, Palliative and End of Life Care
Department	SESLHD
Position Responsible<br>(for ongoing maintenance of Protocol)	SESLHD Stream Director, Palliative and End of Life Care

**GOVERNANCE**	
Enactment date<br>*Reviewed* (Version 2)<br>*Reviewed* (Version 3)<br>*Reviewed* (Version 3.1)	November 2020<br>November 2022<br>December 2023<br>April 2025
Expiry date:	April 2027
Ratification date by SESLHD DTC	3 April 2025
Chairperson, DTC Committee	Stuart Binns, Acting Chair, SESLHD DTC
Version Number	4.0
